# A low balance between microparticles expressing tissue factor pathway inhibitor and tissue factor is associated with thrombosis in Behçet’s Syndrome

**DOI:** 10.1038/srep38104

**Published:** 2016-12-07

**Authors:** E. Khan, N. L. Ambrose, J. Ahnström, A. P. Kiprianos, M. R. Stanford, D. Eleftheriou, P.A. Brogan, J. C. Mason, M. Johns, M. A. Laffan, D. O. Haskard

**Affiliations:** 1 Vascular Sciences Section, National Heart and Lung Institute, Imperial College, London, UK; 2 Centre for Haematology, Department of Medicine, Imperial College, London, UK; 3 Department of Ophthalmology, King’s College, London, UK; 4 Institute of Child Heath, University College, London, UK

## Abstract

Thrombosis is common in Behçet’s Syndrome (BS), and there is a need for better biomarkers for risk assessment. As microparticles expressing Tissue Factor (TF) can contribute to thrombosis in preclinical models, we investigated whether plasma microparticles expressing Tissue Factor (TF) are increased in BS. We compared blood plasma from 72 healthy controls with that from 88 BS patients (21 with a history of thrombosis (Th+) and 67 without (Th−). Using flow cytometry, we found that the total plasma MP numbers were increased in BS compared to HC, as were MPs expressing TF and Tissue Factor Pathway Inhibitor (TFPI) (all p < 0.0001). Amongst BS patients, the Th+ group had increased total and TF positive MP numbers (both p ≤ 0.0002) compared to the Th- group, but had a lower proportion of TFPI positive MPs (p < 0.05). Consequently, the ratio of TFPI positive to TF positive MP counts (TFPI/TF) was significantly lower in Th+ *versus* Th− BS patients (p = 0.0002), and no patient with a TFPI/TF MP ratio >0.7 had a history of clinical thrombosis. We conclude that TF-expressing MP are increased in BS and that an imbalance between microparticulate TF and TFPI may predispose to thrombosis.

Behçet’s syndrome (BS) is a multisystem inflammatory disorder, characterized by oral and genital ulceration, skin lesions, arthritis and uveitis^1^. Venous thrombosis occurs in 10–40% of cases, and is a leading cause of morbidity and mortality in the condition^2^,^3^,^4^. Venous thrombosis particularly occurs in young males with a history of inflammatory eye disease, and is most severe in this group. Immunomodulation is considered to be central to the management of BS patients with thrombotic complications^5^, but the role of anticoagulation is still controversial^6^.

The cause of thrombosis in Behçet’s syndrome remains unknown. A number of studies have looked for changes in the hemostatic and fibrinolytic systems in BS, but no clear abnormality has been adopted for measurement in clinical practice^7^. Consequently, there is a need both for better understanding of the pathophysiology and for biomarkers to aid in clinical management.

Tissue Factor (TF, CD142) is a 47 kDa transmembrane cell surface glycoprotein that triggers the extrinsic coagulation pathway^8^. It is highly expressed by stromal cells within the adventitia of blood vessels, providing a protective ‘haemostatic envelope’ that activates blood coagulation upon vascular injury. However, TF also has a major pathologic role in linking thrombosis with inflammation^9^. On the one hand TF expression is induced by inflammatory activation of monocytes/macrophages by endotoxin or inflammatory cytokines^10^,^11^, and on the other hand the generation of thrombin via TF expression leads to the inflammatory activation of cells via protease-activated receptors^12^.

The importance of circulating microparticles (MP) to disease is increasingly appreciated^13^. Although MP are highly heterogeneous, a population derived from cell plasma membranes can be identified by annexin V binding to surface phosphatidylserine (PS). Circulating annexin V+ MP expressing TF (TF+MP) harbor the majority of TF in blood plasma and have been considered to be primarily derived from monocyte/macrophage membranes^14^,^15^. Not only have TF+ MP been found to contribute to clot propagation in preclinical models^16^,^17^,^18^, but numbers of circulating TF+ MP have been related to clinical thrombosis in sepsis, atherosclerosis, malignancy and venous thromboembolism^19^,^20^,^21^,^22^.

Tissue Factor Pathway Inhibitor (TFPI) is a single chain Kunitz-type proteinase inhibitor that is primarily produced by endothelial cells, although it is also synthesized by other cell types including monocytes^23^,^24^. TFPI acts as an anti-coagulant through inhibiting Factor Xa, and, once bound to Factor Xa, inhibits the Factor VIIa/TF complex^25^,^26^. The majority of TFPI in the circulation is the truncated beta form, linked to the surface of vascular endothelium by a glycophosphatidylinositol anchor^27^. The full length alpha form also binds endothelial cells, probably via glycosaminoglycans, and circulates in plasma bound to lipoproteins^28^. However TFPI may be also detected on some circulating MP^29^,^30^. Low overall plasma levels of TFPI have been associated with recurrent venous thrombosis in the general population^31^. A previous study observed increased levels of plasma TFPI in BS patients overall, although a difference between those with and without a history of thrombosis was not addressed^32^. The amount of TFPI that can be mobilized into the circulation by heparin infusion has been found to be reduced in BS, suggesting that TFPI associated with endothelium is reduced and raising the possibility that this might confer a prothrombotic risk^33^.

Quantities of plasma MP are known to be increased in BS, but previous studies these have not addressed an association between MP and thrombosis^34^,^35^. We investigated the hypothesis that numbers of plasma MP expressing TF are increased in BS. We found that this is the case, and that patients with a low ratio of MP counts expressing TFPI to MP counts expressing TF are significantly more likely to have a history of thrombosis.

## Results

### Study population

**Figure 1:**
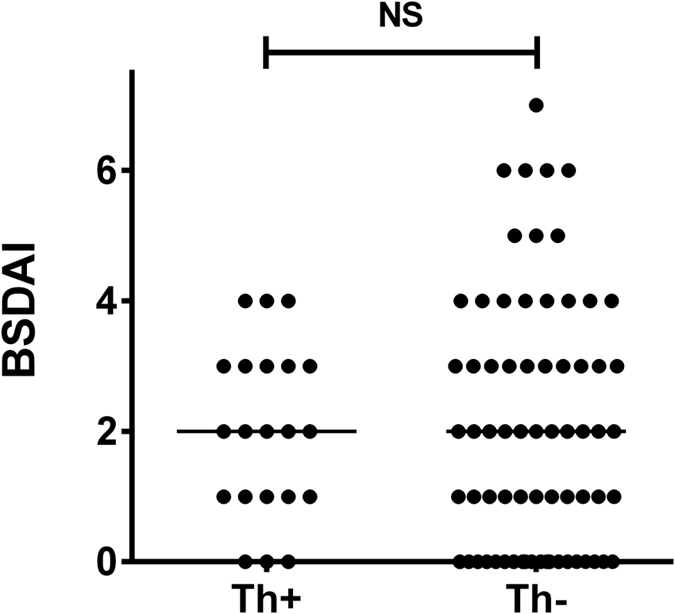
Scoring on the Behçet’s Syndrome Disease Activity Index (BSDAI) of BS patients in the study, comparing patients with (Th+) and without (Th−) a history of thrombosis. There was no significant difference between the two groups.

The demographic and clinical details of the 88 BS patients and the 74 age- and sex-matched HC in the study are shown in Table 1. Current BS disease activity was low (median BSDAI score 2 out of a maximum of 12) with no difference between those with (Th+) or without (Th−) a history of thrombosis (Fig. 1). Within the BS group, 21 (24%) had a history of thrombosis, including 13 lower limb deep vein thromboses, 11 retinal vein occlusions (8 branch retinal vein and 3 central retinal vein), 2 superior vena cava occlusions and 1 dural sinus thrombosis. Six patients had had more than one thrombotic episode. Three patients had been told that they had suffered a pulmonary embolus, two of whom had a previous deep vein thrombosis and one a dural sinus thrombosis.

### Quantification of annexin V positive MP

**Figure 2:**
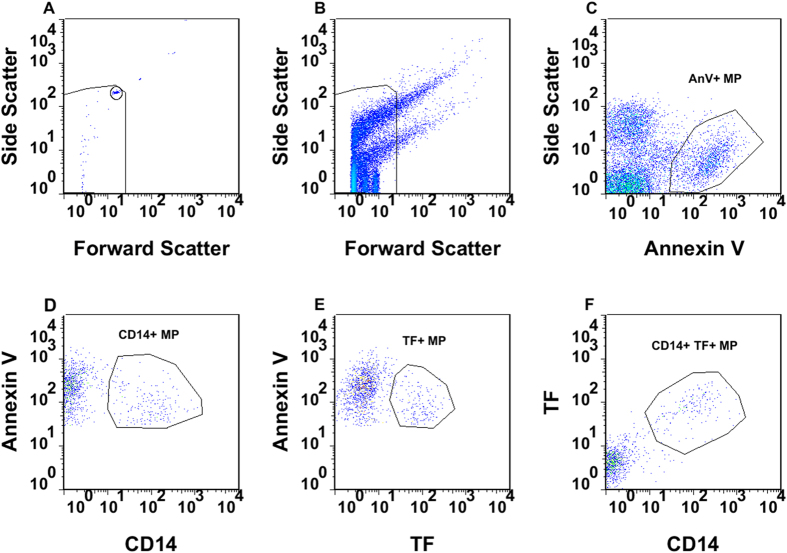
Gating strategy for flow cytometry. All MP were gated <1.1 μm in size using microbeads. **(A,B)** Forward and side scatter of (**A**) 1.1 μm microbead suspension and (**B**) plasma microparticles ; **(C)** particles <1.1 μm binding annexin V (AnV+) were defined as MP; **(D,E)** gating of annexin V positive MP positive for (**D**) CD14 or (**E**) TF; **(F)** annexin V positive MP stained by both anti-CD14 and anti-TF.

**Figure 3:**
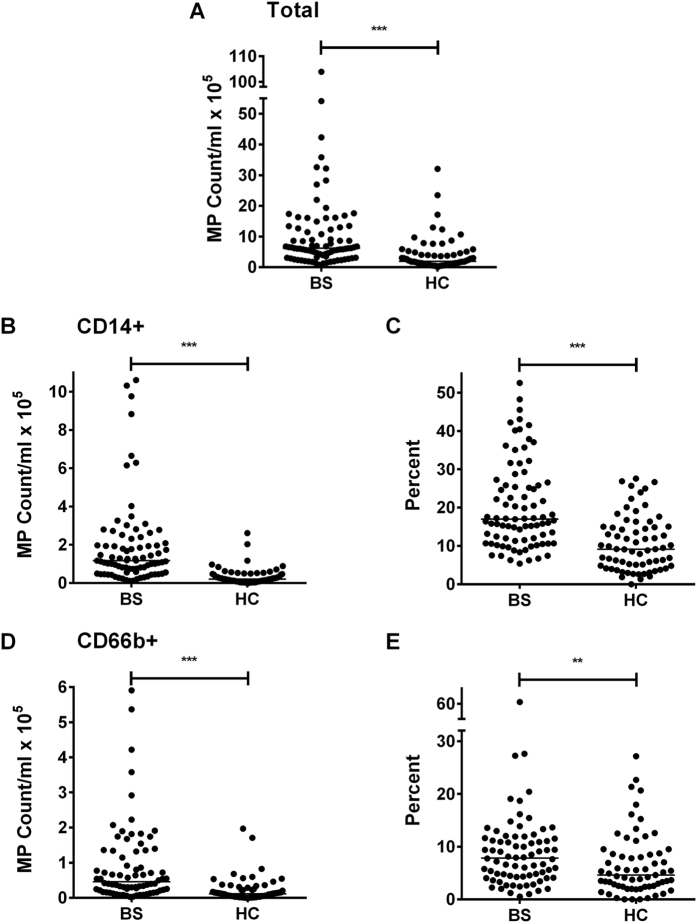
Behçet’s Syndrome (BS) patients have higher MP counts than HC. MP were enumerated in BS *versus* HC plasma by flow cytometry using annexinV and mAb against CD14 and CD66b. **(A)** total MP; **(B,C)** CD1+ MP; **(D,E)** CD66b+ MP. Data are expressed as absolute counts **(A,B,D)** or % total MP **(C,E)**. **p < 0.01; ***p < 0.001.

We defined and enumerated plasma MP using flow cytometry by gating on particle size (<1.1 μm), reactivity with annexin V and with specific antibodies (Fig. 2, Supplementary Figure 1). As shown in Fig. 3A, BS patients had an increased absolute total MP count compared to HC (medians: 6.18 × 10^5^/ml versus 1.90 × 10^5^/ml; p < 0.0001). By staining with antibodies, we found that the number of CD14 positive MP (CD14+ MP) was also increased, either when expressed as absolute number of CD14+ MP (medians: 1.17 × 10^5^/ml versus 2.05 × 10^4^/ml; p < 0.0001; Fig. 3B), or as a percentage of total MP (medians: 16.97% versus 9.18%; p < 0.0001; Fig. 3C). This suggests that the increased MP in BS derive at least in part from monocytes and monocyte-derived macrophages, although neutrophils also express CD14 at low density and may release TF+ MP^36^,^37^. An increase in granulocyte-derived MP in BS was indeed suggested by an increase in the absolute number (p < 0.0001; Fig. 3D) and percentage (p < 0.01; Fig. 3E) of CD66b+ MP. The total of CD14+ and CD66b+ MP did not account for all the MP, but the origins of the remainder were not analysed.

### Quantification of MP expressing Tissue Factor

**Figure 4:**
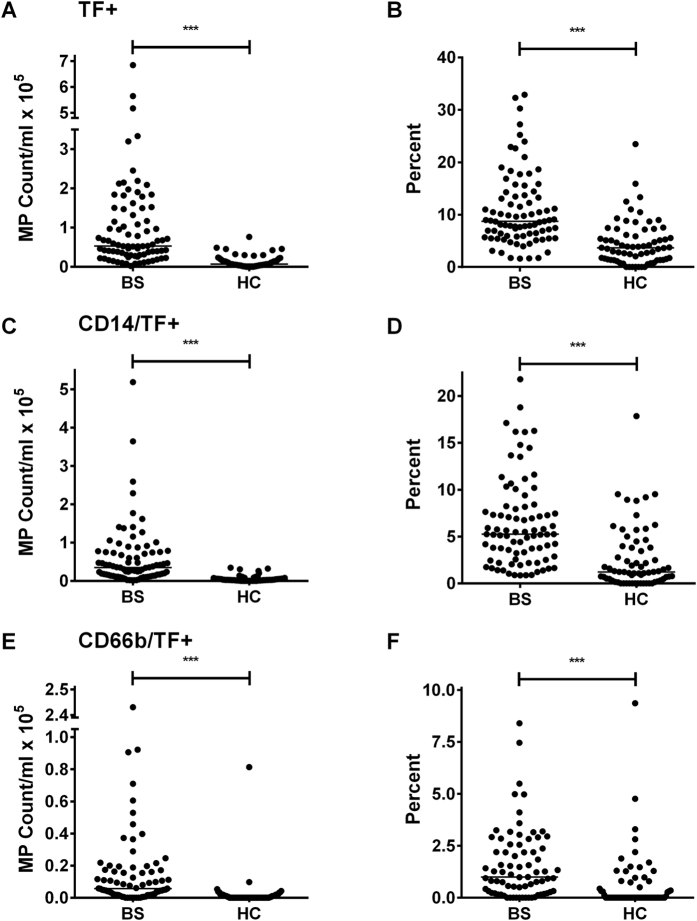
Behçet’s Syndrome (BS) patients have higher numbers of TF+ MP than HC. MP were enumerated in BS *versus* HC plasma by flow cytometry using annexin V and mAb. **(A,B)** total TF+ MP; **(C,D)** CD14/TF+ MP; **(E,F)** CD66b/TF+ MP. Data are expressed as absolute counts **(A,C,E)** or % total MP **(B,D,F)**. ***p < 0.001.

By costaining with anti-TF monoclonal antibody (Fig. 2E), we determined whether numbers of TF+ MP are also increased in BS. This was the case, with both an increase in absolute number (medians: 5.31 × 10^4^/ml versus 6.70 × 10^3^/ml; p < 0.0001; Fig. 4A) of TF+ MP and the percentage of total MP that were TF positive (medians: 8.72% versus 3.66%; p < 0.0001; Fig. 4B). By triple staining using anti-CD14 (Fig. 4C,D) or CD66b (Fig. 4E,F), we were able to confirm that the increase in TF+ MP in BS patients related at least in part to MP release by cells of the monocytic and/or granulocyte lineage. Differences between BS patients and HC remained after smokers in the BS patient group had been excluded (Supplementary Figure 2). Sub-analysis showed no correlation between MP numbers and increasing age (Supplementary Figure 3). Furthermore, sub-analysis of the BS group did not reveal an effect of corticosteroid treatment on MP numbers (Supplementary Figure 4). Further sub-analyses to relate MP numbers to other individual drugs did not reveal any associations.

### Sub-analysis of MP in BS patients according to history of thrombosis

**Figure 5:**
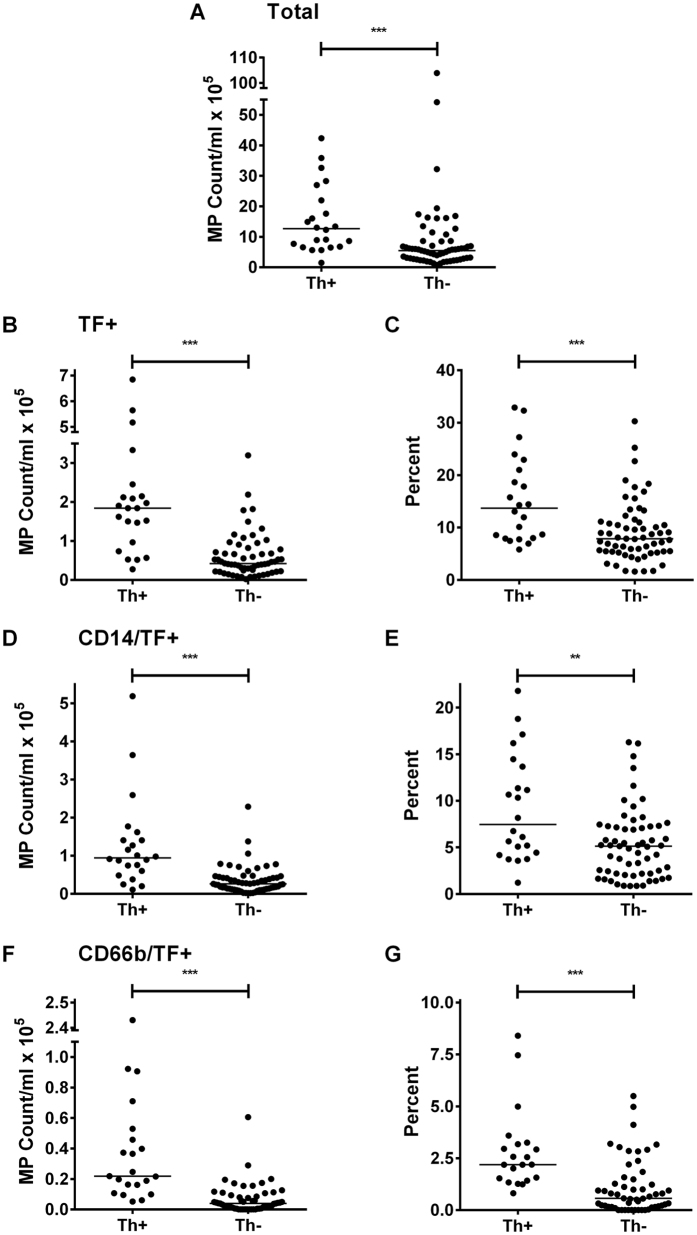
Behçet’s Syndrome (BS) patients with a history of thrombosis (Th+) have higher numbers of TF+ MP than those without (Th−). MP were enumerated in Th+ and Th− BS patients by flow cytometry using annexin V and mAb. (**A**) total MP; (**B,C**) total TF+ MP; (**D,E**) CD14/TF+ MP; (**F,G**) CD66b/TF+ MP. Data are expressed as absolute counts (**A,B,D,F**) or % total MP (**C,E,G**). **p < 0.01, ***p < 0.001.

Compared to BS patients without thrombosis (Th−), BS patients with a history of thrombosis (Th+) had a significantly increased absolute number of MP (p < 0.0001; Fig. 5A), as well as increased TF+, CD14/TF+, and CD66b/TF+ MP counts (Fig. 5B,D,E,G) and percentages (Fig. 5C,E,G), MP numbers were found to be increased in male compared to female BS patients, and this was shown to be due to males having a greater number of Th+ patients (Supplementary Figures 5 and 6). Differences between Th+ and Th− BS patients remained after smokers in the Th+ group were excluded (supplementary Figure 7).

### Quantification of MP-induced thrombin generation

**Figure 6:**
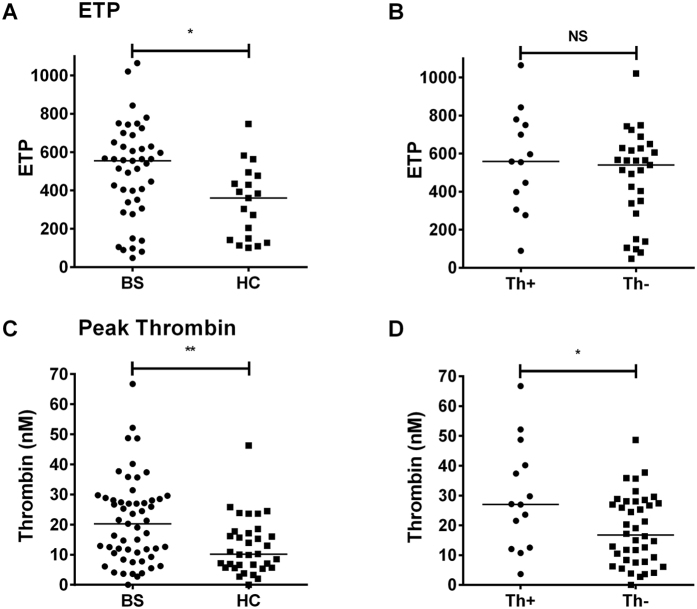
Effects of plasma samples on thrombin generation *in vitro*. Endogenous thrombin potential (ETP) **(A,B)** and peak thrombin **(C,D)** were calculated for (**A,C**) BS compared with HC and (**B,D**) Th+ compared with Th− groups. * p < 0.05, **p < 0.01, NS = non-significant.

Using calibrated automated thrombography (CAT), we found that thrombin generation due to PPP was too low for comparisons to be made. We therefore went on to use five-fold concentration of microparticles suspended in a standard MPPP pooled from HC. Despite this, there were 14 BS samples that did not generate thrombin (1 from a Th+ patient and 13 from a Th− patient), and these were not included in the final analysis. We found a significantly increased endogenous thrombin potential (ETP) and peak thrombin generation in the BS group compared to HC (p < 0.05 and <0.01, respectively) (Fig. 6A,C), but no difference between groups in lag time to thrombin generation (not shown). Within the BS group, patients with a history of thrombosis had a significantly increased peak thrombin compared to those without (Fig. 6D; p < 0.05), but there was no difference between these groups in ETP (Fig. 6B) or lag time (not shown). Comparing samples that did not generate thrombin between groups, there was no significant difference found between Th+ and Th− BS populations (not shown). Using Pearson correlation coefficient, we were unable to demonstrate significant correlations between MP counts and any index of thrombin generation *in vitro* (not shown).

### Quantification of MP expressing Tissue Factor Pathway Inhibitor

**Figure 7:**
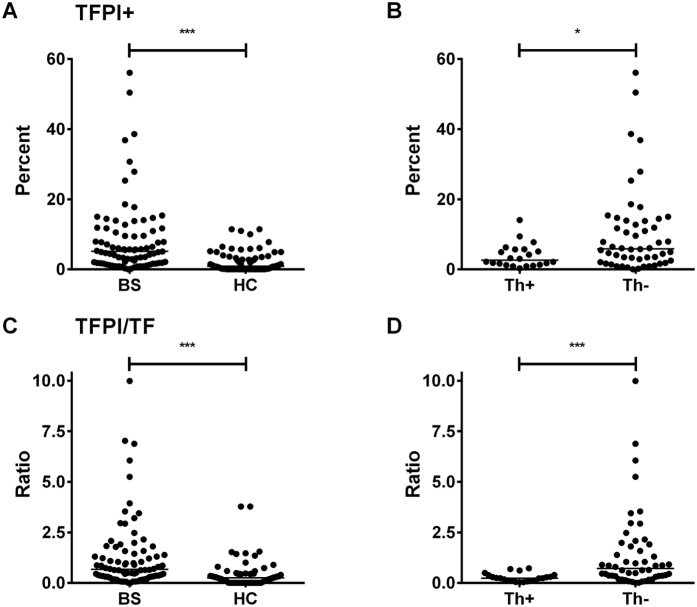
Circulating TFPI+ MP are increased in BS but relatively low in patients with a history of thrombosis. (**A**,**B**) show TFPI+ MP in (**A**) BS *versus* HC and (**B**) Th+ *versus* Th− BS patients expressed as % total MP counts; **(C,D)**; show ratios of TFPI to TF counts in (**C**) BS *versus* HC and (**D**) Th+ *versus* Th− patients.

The failure of the functional assay to show a better relation to TF+ MP numbers might at least in part be explained by variable expression of an inhibitor such as TFPI. As judged by flow cytometry (Supplementary Figure 8), BS patients as a whole had a higher absolute number (medians: 3.60 × 10^4^/ml versus 2.00/ml; p < 0.0001; not shown) and percentage (medians: 5.17% versus 0.91%; p < 0.0001; Fig. 7A) of TFPI+ MPs, indicating that an increase in TFPI+ MPs in the disease environment may be a protective response. The absolute number of TFPI+ MPs did not significantly differ between Th+ and Th− BS patients (not shown), but, surprisingly, we found that a significantly lower percentage of MP were TFPI+ in the BS patients with a history of thrombosis (Fig. 7B; p < 0.05). We calculated the ratio of TFPI+ MPs to TF+ MPs in BS vs HC (Fig. 7C) and Th+ vs Th− (Fig. 7D). BS patients overall had a significantly higher median ratio than HC (0.67 *vs*. 0.25; p < 0.0001). Strikingly, Th− BS patients without a history of thrombosis had a higher median TFPI/TF MP ratio compared with Th+ patients (0.72 *vs.* 0.23; p < 0.0001), with no patient with a ratio of >0.7 having a history of a thrombotic event.

## Discussion

We believe that our data add significantly to the understanding of thrombosis in BS. We found that BS patients not only have significantly higher numbers of MP compared to healthy controls, but also have higher numbers of MP expressing TF. Furthermore, BS patients with a history of thrombosis had higher TF+ MP numbers than those without a thrombosis history. Based on costaining of MP with antibodies against CD14 and CD66, it is likely that TF+ MP derive at least in part from cells of monocyte and/or granulocyte lineage, although other cell types probably also contribute to the circulating TF+ MP pool. The release of TF+ MP by activated monocytes and/or granulocytes provides a plausible link between inflammation and thrombosis in BS.

The population of MP in plasma is highly heterogeneous and there is ongoing debate as to how samples should be prepared and how MP should best be measured^38^. Many laboratories perform a centrifugation step to concentrate MP for analysis^39^, and we decided to centrifuge PPP for an hour at 17,000 G, avoiding ultracentrifugation to minimize aggregation. MP were detected by flow cytometry, which is the most common technique used and is more applicable to the clinical setting than others such as atomic force microscopy or nanoparticle tracking analysis. Our decision to use flow cytometry on centrifuged PPP and to focus on annexin V+ MP detectable within a defined forward and side scatter gate set with the use of 1.1 μm latex beads was pragmatic and based on the need to consistently process a reasonably large number of samples and to also obtain data on surface antigens. Two other technical considerations should be noted. First, that we used the unstained MP suspension as the control for annexin V binding, and any background non-specific binding might have been lower by incorporating annexin V in calcium chelating buffer. Secondly, we recognize that differences in the refractive index of MP compared to latex beads may cause difficulties in the precise sizing of the MP we have studied^40^.

Our analysis was confined to the Annexin V positive MP population falling within our forward and side scatter gating, and analyzing Annexin V negative MPs in Behçet’s syndrome may also be informative^41^,^42^. However lipoproteins may also fall within the Annexin V negative population^39^. For practical reasons, many of our patients were not fasting, and so we decided not to assess the Annexin V negative data in the present study.

We initially tested the thrombin generating capacity of BS samples using PPP, but found that the amount of thrombin generated was insufficient to be useful. Consequently, we adapted the assay by testing five-fold concentrates of MP suspended in a standard MPPP that had been pooled from HC. Apart from focusing on the MP fraction, this also enabled us to minimize the effects that any warfarin treatment might have had on coagulation factor concentrations in patient plasma. The results showed that MP-stimulated thrombin generation was relatively poor at differentiating either BS from HC samples or Th+ from Th− samples. This is consistent with many previous studies that have failed to find standard coagulation assays useful in this setting. The lack of correlation between the functional assay and numbers of MP expressing both PS and TF is in keeping with the TF that is recognized on MP by immunostaining being mostly latent rather than active, and it is possible that MP-associated TF only becomes functional in the local milieu of the vessel wall at sites of vascular inflammation once MP are caught by fibrin or neutrophil extracellular traps^17^. The activation of trapped MP-associated TF by disulphide isomerase released locally may then increase the probability of clot propagation and overt thrombosis, as happens in preclinical models^16^,^17^,^18^. A further mechanism enhancing clot propagation in BS may be the recently described oxidative injury to fibrinogen, which makes clots more resistant to lysis^43^.

The poor correlation between TF+ MP and the thrombin generation assay may also be explained by the variable presence of TFPI^44^. Our observation that numbers of circulating MP expressing TFPI were significantly increased in BS compared to HC is consistent with a previous report measuring total plasma TFPI by ELISA, and this may be a reactive homeostatic adaptation that serves to reduce the probability of thrombosis in the inflammatory setting^32^. On this background, it was particularly interesting that BS patients with a history of thrombosis had a significantly lower proportion of TFPI+ MP compared to those without. We do not know whether TF and TFPI are expressed by the same or different MPs, but, notwithstanding, the implication of this observation is that the balance between MP-associated TF and TFPI may determine the risk of thrombosis. This is supported by Th− patients having a significantly higher TFPI/TF ratio, and indeed by the absence of a thrombosis history in all individuals with a ratio >0.7. The reasons behind some BS patients having a relatively low level of MP-associated TFPI compared to MP-associated TF are unclear, but could include differential synthesis of TF and TFPI by activated monocytes^24^, TFPI degradation by leukocyte-derived serine proteases and/or reactive oxygen species^45^,^46^ and the influence of genetic variants of TFPI on plasma levels^47^.

The retrospective nature of our study is an obvious limitation. There is a small possibility that the differences in MP counts that we have detected in the Th− patients followed rather than preceded the thrombotic event. This seems unlikely, as in the majority of patients the interval between thrombosis and MP testing was greater than a year. It would be ideal to conduct a prospective study testing the ability of TF+ and TFPI+ MPs to predict a first thrombosis in BS. However such a project would be difficult to perform, both because thrombosis is often the presenting manifestation, and because treatment has often commenced prior to referral to a specialist centre^48^,^49^. Although our study is relatively large for a BS study in the UK, it will be important to substantiate the generalizability of our observations on larger groups from endemic regions with more patients, such as Turkey, the Middle East, Korea and Japan.

The current mainstay of treatment of thrombosis in BS is immunomodulation, either using disease-modifying agents such as azathioprine, or biologic agents such as tumor necrosis factor alpha inhibitors. Anticoagulants alone do not appear to be effective in preventing recurrence. Thus, a small trial in Korea found that thrombosis recurred in three out of four patients treated with anti-coagulation alone, but that immunosuppression, either with or without anticoagulation, was associated with reduced recurrence of thrombosis^50^. Similarly, two larger retrospective studies from France and Turkey have concluded that immunosuppression is associated with reduced thrombosis relapse^49^,^51^.

In conclusion, our study cuts new ground by demonstrating that BS patients may have high circulating levels of MP expressing TF and that these are more numerous in patients with a history of thrombosis. Furthermore, discrimination between patients with and without a history of clinically overt thrombosis was improved by factoring in the level of MP expressing TFPI. The balance between TF+ and TFPI+ MPs may prove useful in thrombosis risk assessment and in making decisions to initiate or withdraw immunosuppressive treatment.

## Methods

### Ethics

Approval for the study was obtained from the Hammersmith, Queen Charlotte’s and Chelsea Hospitals Research Ethics Committee (ref 2001/6179) and all methods were performed in accordance with the relevant guidelines and regulations. All participants gave written informed consent to participate.

### Behçet’s syndrome patients and healthy controls

This was a cross-sectional study of 88 BS patients and 74 age- and sex-matched healthy controls (HC), all over 18 years old. Patients were recruited from specialist clinics at the Hammersmith and St Thomas’ hospitals in London over a period from 2012–2015. The BS patients fulfilled the 1990 International Study Group diagnostic criteria^52^. Specific patient history included BS manifestations, with details of thrombotic episodes. BS disease activity was quantified using the Leeds Behçet’s Syndrome Disease Activity Index (BSDAI)^53^. A medication and smoking history was taken from all participants. Healthy controls were free of known disease and not taking medication, and were selected to provide for age and sex-matched comparisons. Laboratory analyses were conducted blind of the identity of plasma donor to avoid bias.

### Blood sampling

Peripheral blood was taken from participants using a 21 G needle in accordance to local aseptic non-touch technique guidelines. The first 5 ml were either utilized for serological analyses or were discarded, depending on whether the participant was a BS patient or HC respectively, and 20 mls were transferred into a 50 ml Falcon tube containing 2 ml 3.2% sodium citrate (Sigma, St. Louis, USA).

### Platelet poor plasma preparations

Citrated peripheral blood was processed to form platelet poor plasma (PPP) within 1–2 hours of venesection. In the intervening period, care was taken to avoid inverting the sample that could inadvertently lead to the increased production of MP. Samples underwent two 5000 g × 10 min centrifugation steps. After each step, plasma was decanted, being careful not to contaminate it with the pellet. The final PPP preparation was stored as 500 μl aliquots at −80 °C.

### MP preparation

Aliquots of PPP were thawed in a 37 °C water bath for 1–2 mins. This was done to prevent the development of cryoprecipitates that would impact on analysis^54^. The aliquots were then centrifuged for 1 hour at 17,000 g at 4 °C to sediment MPs. Subsequently, 480 μl of supernatant was retained as MP poor plasma (MPPP), leaving 20 μl containing the MP pellet. This was resuspended to its original 500 μl volume using Annexin V buffer (BD Pharmingen, Oxford, UK) and stored on ice ready for flow cytometry. For calibrated automated thrombography (CAT), 490 μl of MPPP was retained leaving 10 μl containing the MP pellet ready for use.

### Flow cytometry reagents

Reagents used for flow cytometry were: Pacific Blue (PB) conjugated annexin V (Biolegend, San Diego, USA), fluorescein isothiocyanate (FITC) conjugated anti-tissue factor (CD142) (Abd Serotec, clone CLB/TF-5, Oxford, UK), phycoerythrin (PE) conjugated anti-CD14 (Invitrogen, clone Tük4) and PE conjugated CD66b (Biolegend, clone G10F5, San Diego, USA). Murine anti-I K1 monoclonal antibody (Sanquin, clone CLB/TFPI Kunitz domain 1 specific, Amsterdam, Netherlands) was conjugated to AF-647 Apex^®^ molecular beads (Invitrogen Life Sciences, Paisley, UK). The isotype control antibodies were FITC conjugated IgG1 (Abd Serotec, Oxford, UK), PE conjugated IgG1 (Invitrogen, Paisley, UK), PE conjugated IgM_ĸ_ (Biolegend, San Diego, USA) and murine IgG1 (Sigma, St. Louis, USA) for binding to AF647 Apex^®^ molecular beads.

### Microparticle labelling

The MP suspension (40 μl) was incubated with PB-conjugated annexin V and then co-stained with either PE-labelled anti-CD14 plus FITC-labelled anti-TF, or PE-labelled anti-CD66b plus FITC-labelled anti-TF, or AF-647 conjugated anti-TFPI K1 antibody only. To assist with gating, an unlabeled sample and annexin V buffer was used, as well as the appropriate isotype controls. MPs were incubated at room temperature on a shaker for 30 min in the dark, and the reaction was stopped with the addition on 200 μl annexin V buffer. All flow cytometry reagents were tested and titrated prior to analysis of BS and HC samples. Importantly, PB-annexin V and all conjugated antibodies were initially run in annexin V buffer only to make sure that there was no signal from these reagents, and none was found.

### Flow cytometry

A standardized protocol was used to ensure reproducibility and reliable quantification of samples. MPs were identified by forward and side scatter, and by binding of annexin V. Latex beads (1.1 μm diameter; Sigma, St. Louis, USA) were used to confirm MP were below this size, whilst a known concentration of 3 μm latex beads (Sigma, St. Louis, USA) (200,000/ml) was used to enumerate MP during 30 second runs. To minimize day-to-day flow cytometer variability, this procedure was used before measurement of all samples. Gates setting specific staining with each marker compared to isotype-matched control antibody are illustrated in Supplementary Figure 1. MP counts were calculated using equation 1:


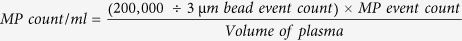


### Thrombin generation assay

The thrombotic potential of MP was assessed using CAT, which has previously been used to assess the thrombotic potential of MP in a number of conditions^55^,^56^,^57^. To assay PPP, 80 μl with 20 μl buffer were recalcified by addition of CaCl_2_ and fluorescent substrate Z-Gly-Gly-Arg-AMC (Bachem, Bubendorf, Switzerland), to a final concentration of 16.6 mM and 0.42 mM respectively, in a total volume of 120 μl. To assay MP, 10 μl concentrate of MP was added to 80 μl MPPP (pooled from 10 HC), 10 μl buffer and recalcified by the addition of CaCl_2_ and fluorescent substrate, as with PPP samples. Thrombin generation from PPP and MP was followed in real time using a Fluoroscan Ascent FL plate reader (Thermo Labsystem, Paisley, UK) and Thrombinoscope software (Synapse BV, Maastricht, Netherlands). Data gathered included: endogenous thrombin potential (ETP), which represents the area under the curve and total thrombin generated; peak thrombin; lag time to thrombin generation. Samples were run in duplicate and only those samples that generated thrombin were included in the analysis.

### Statistics

As this was an exploratory study, the numbers of BS patients and healthy controls were arrived at empirically on the basis of availability of BS patients. Results of all the assays followed a non-Gaussian distribution and so are expressed as medians. GraphPad PRISM 6.07 (GraphPad Software, Inc, San Diego, CA, USA) was used for statistical analyses and graph generation. Statistical comparisons of median MP counts, MP proportions and CAT assay measures were accomplished using Mann-Whitney U test with significance set at p < 0.05.

## Additional Information

**How to cite this article**: Khan, E. *et al*. A low balance between microparticles expressing tissue factor pathway inhibitor and tissue factor is associated with thrombosis in Behçet’s Syndrome. *Sci. Rep.* **6**, 38104; doi: 10.1038/srep38104 (2016).

**Publisher's note:** Springer Nature remains neutral with regard to jurisdictional claims in published maps and institutional affiliations.

## Supplementary Material

Supplementary Figures 1–8

## Figures and Tables

**Table 1:** Details of patients and controls.

	Healthy Controls		Behçet’s Syndrome	
*General Demographics*				
Cohort size	74		88	
Median age (range)	41	(18–75)	43	(17–74)
Male sex (%)	40	54%	47	53%
Current smoker	6	8%	12	14%
*Clinical Characteristics*				
Mucocutaneous	—		88	100%
Erythema nodosum	—		29	33%
Pathergy/poor wound healing	—		48	55%
Musculoskeletal - arthralgias	—		71	81%
Arthritis	—		30	34%
Vascular	—		22	25%
Aneurysm	—		7	8%
Thrombosis	—		21	24%
Posterior/panuveitis	—		39	44%
Gastrointestinal inflammation	—		10	11%
Neurological	—		18	20%
Median BSDAI*	—		2	
*Current medications*				
Colchicine	—		22	29%
Azathioprine	—		15	19%
Mycophenolate	—		12	16%
Anti-TNF therapy	—		16	21%
Cyclosporine	—		3	4%
Methotrexate	—		9	12%
Steroids†	—		24 (7.5 mg)	31%
Warfarin	—		12	14%

^*^Behçet’s Syndrome Disease Activity Index = BSDAI.

^†^Median dose in brackets.
