# Supplementary material for: A low balance between microparticles expressing tissue factor pathway inhibitor and tissue factor is associated with thrombosis in Behçet’s Syndrome
Source: Sci Rep. 2016 Dec 7;6:38104. doi: 10.1038/srep38104 (PMC5141484; doi:10.1038/srep38104)
Supplement: Supplementary Figures 1–8 [file srep38104-s1.pdf]

A low balance between microparticles expressing tissue factor pathway inhibitor and tissue factor is associated with thrombosis in Behçet's Syndrome

E.Khan*, N.L.Ambrose*, J.Ahnström†, A.P.Kiprianos*, M.R.Stanford‡, D.Eleftheriou§, P.A. Brogan§, J,C.Mason*, M.Johns*, M.A.Laffan†, D.O.Haskard *

SUPPLEMENTARY FIGURES

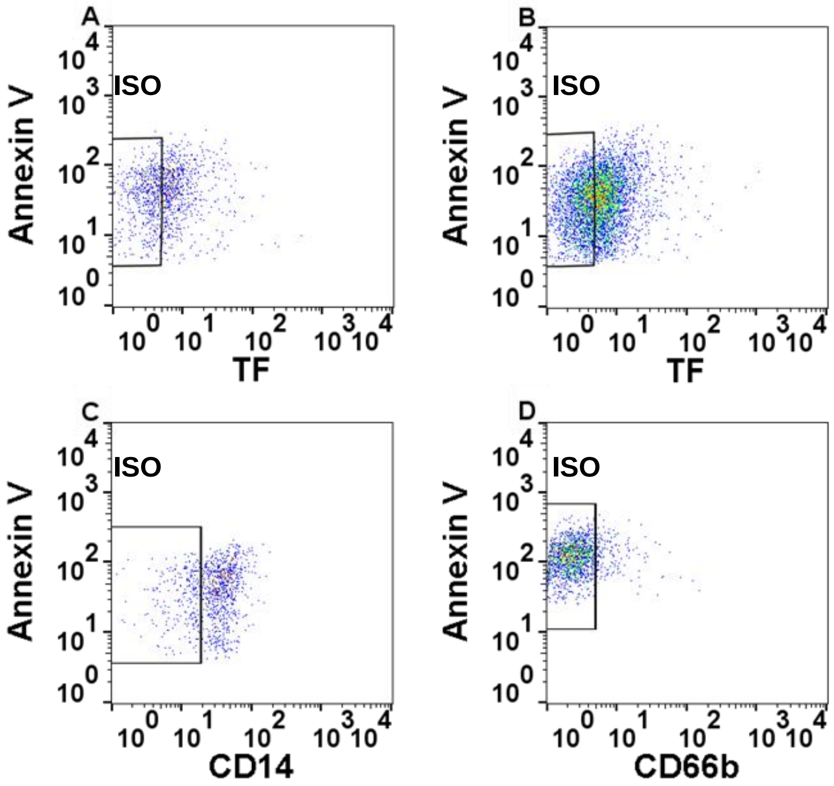

Supplementary Figure 1: Gating strategy to quantify specific TF, CD14 and CD66b staining of Annexin V+ MP relative to non-specific staining with isotype-matched control antibodies. **(A,B)** shows TF staining of MP released into supernatant from unstimulated monocytes **(A)** or from monocytes stimulated with LPS to increase TF expression **(B); (C)** shows CD14 staining of MP released into supernatant from cultured monocytes; (D) shows CD66b staining of plasma MP. The boxes labelled ISO are the gates of non-specific staining excluded from quantification.

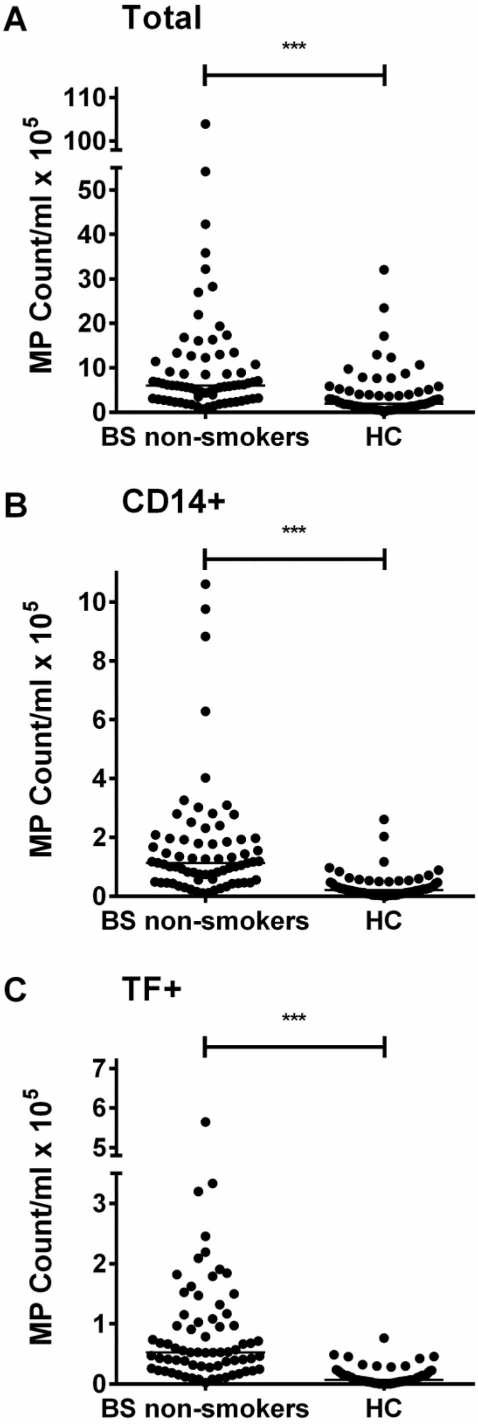

Supplementary Figure 2: Non-smoking Behçet's Syndrome (BS - smokers) patients have higher total **(A)**, CD14+ **(B)** and TF+ **(C)** MP counts than healthy controls (HC). MP were enumerated in non-smoking BS *versus* HC plasma by flow cytometry using annexin V and mAb against CD14 and TF. ***p< 0.001.

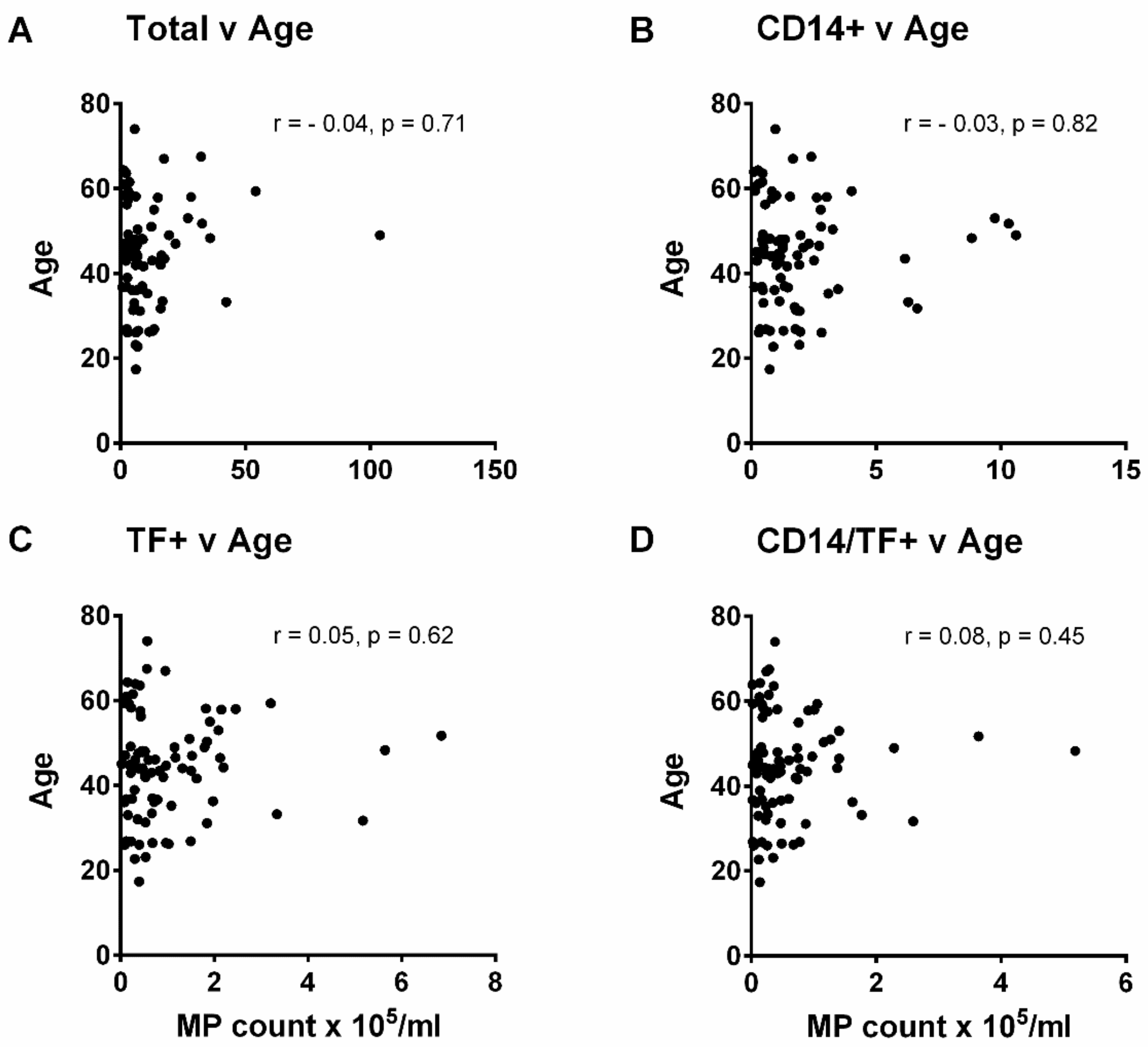

Supplementary Figure 3: Correlation of total **(A)**, CD14+ **(B)**, TF+ **(C)** and CD14/TF+ **(D)** MP counts in BS patient with age. There was no statistically significant correlation found.

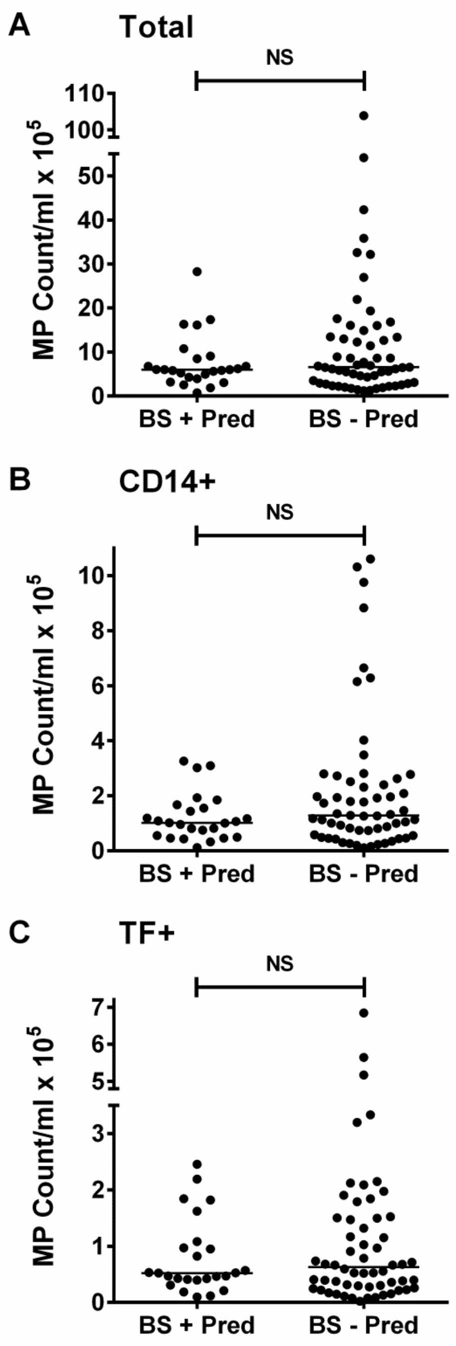

Supplementary Figure 4: The total **(A)**, CD14+ **(B)** and TF+ **(C)** MP counts in Behçet's Syndrome (BS) patients taking prednisolone (BS + Pred) are not significantly different from those in BS patients not taking prednisolone BS – Pred). MP were enumerated by flow cytometry using annexin V and mAb against CD14 and TF. NS = not significant.

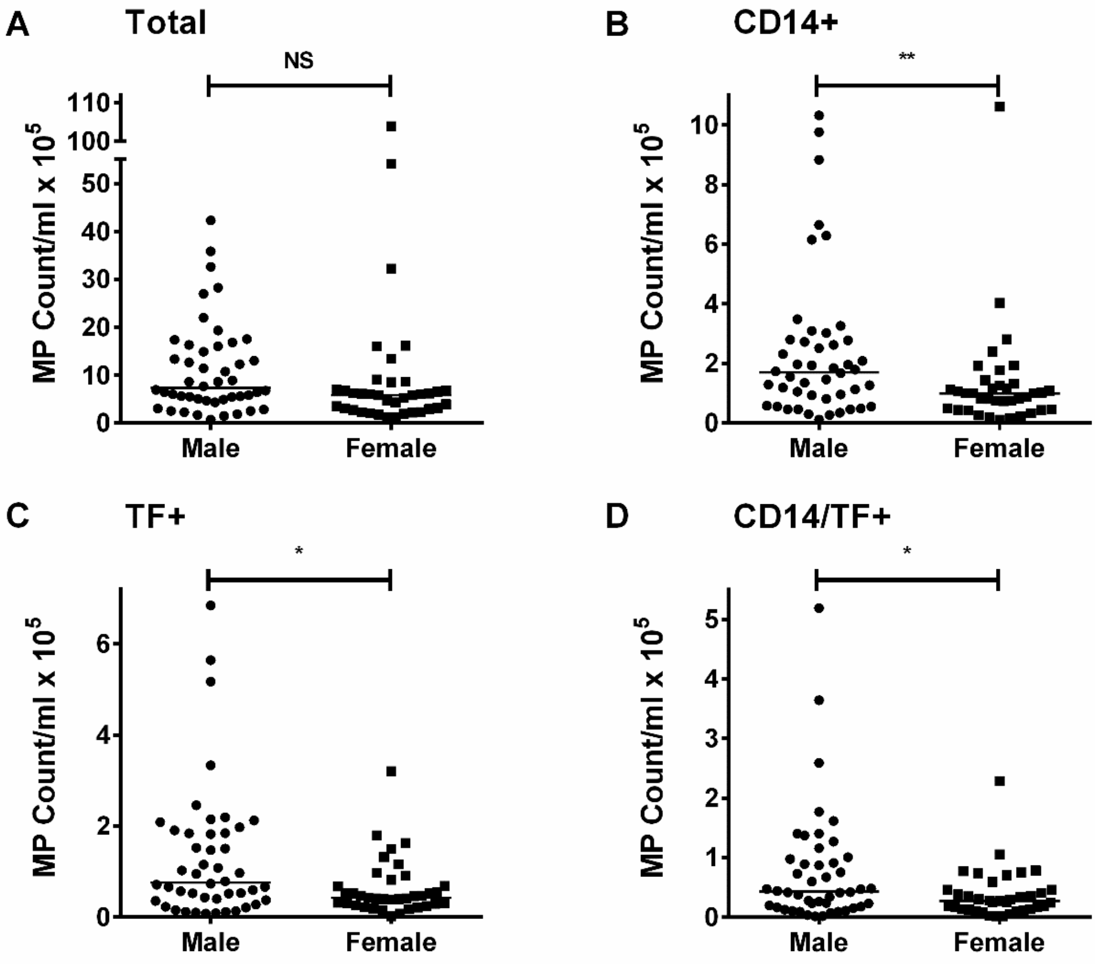

Supplementary Figure 5: MP counts in male and female BS patients. There was no difference in total MP counts **(A)** between males and females, whilst there was significantly higher CD14+ **(B)**, TF+ **(C)** and CD14/TF+ **(D)** MP counts in males compared with females. NS = non-significant, * $p < 0.05$, ** $p < 0.01$.

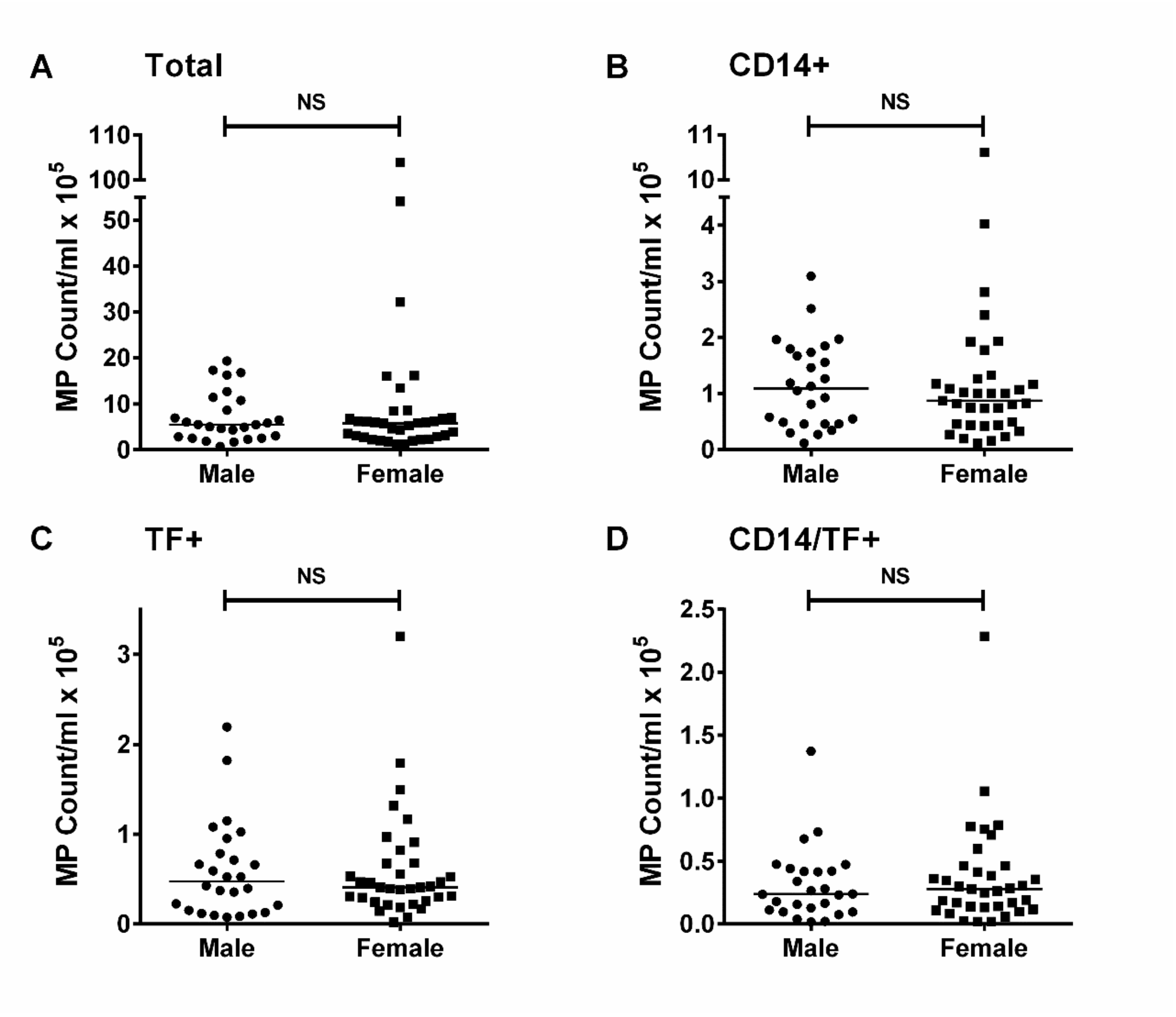

Supplementary Figure 6: MP counts in BS patients without a history of thrombosis, comparing male and females. There was no difference in total **(A)**, CD14+ **(B)**, TF+ **(C)** and CD14/TF+ **(D)** MP counts between males and females. NS = non-significant.

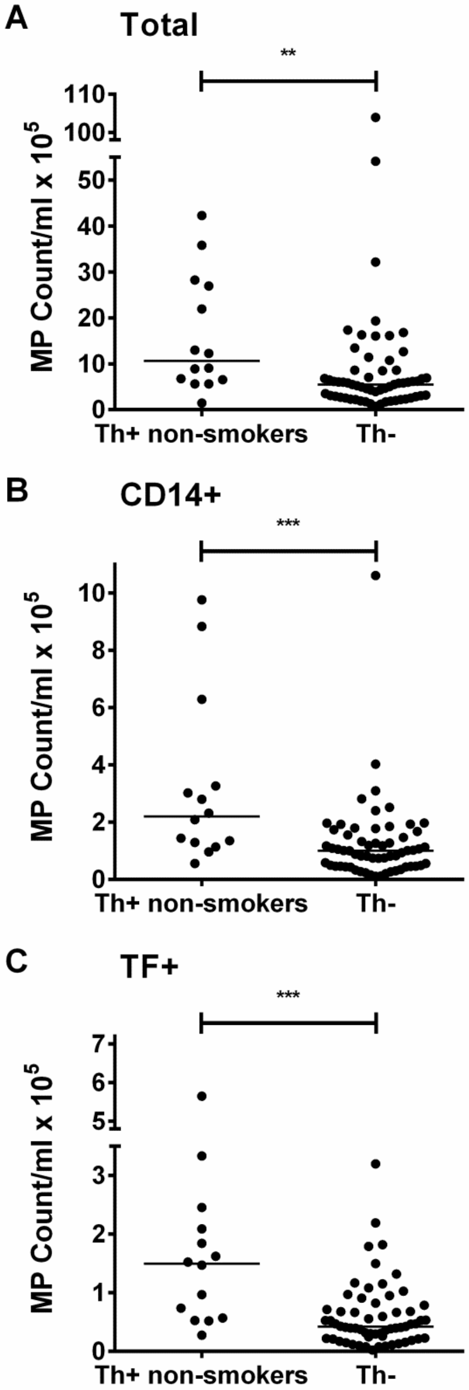

Supplementary Figure 7: Non-smoking Behçet's Syndrome patients with a history of thrombsis (Th+) have higher **(A)** total, **(B)** CD14+ and **(C)** TF+ MP counts than all BS patients without a history of thrombosis. MP were assessed by flow cytometry using annexin V and mAb against CD14 and TF. ** $p < 0.01$, ***$p < 0.001$.

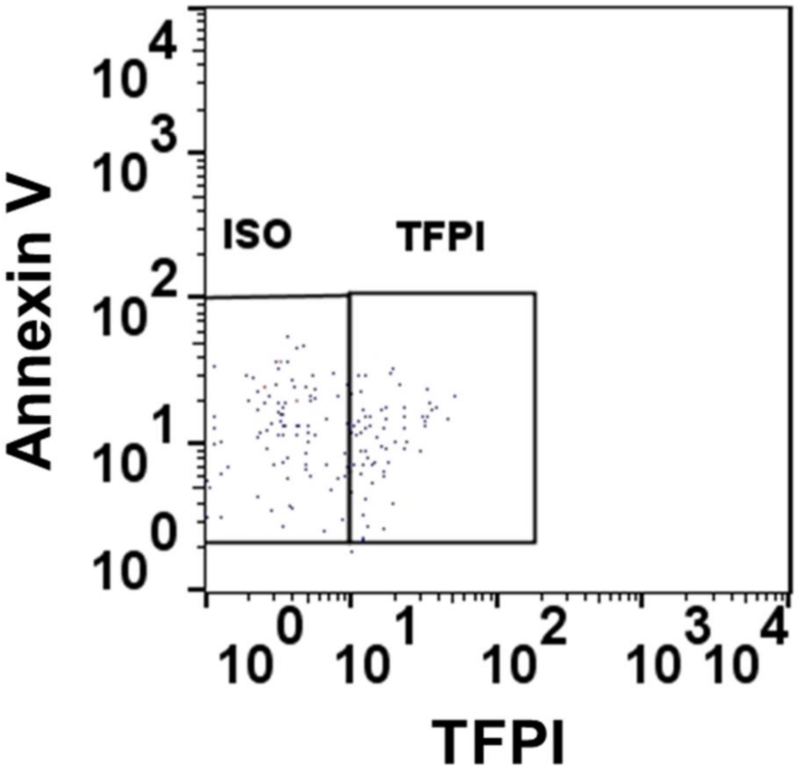

Supplementary Figure 8: MP were enumerated by flow cytometry using annexinV and anti-TFPI mAb. ISO = gating of isotype-matched negative control antibody.
